# IFSMT-based supervised nursing combined with moderate-intensity exercise training for pulmonary rehabilitation in stable COPD: a randomized controlled trial

**DOI:** 10.3389/fpubh.2026.1879076

**Published:** 2026-07-01

**Authors:** Keke Wu, Jing Yang, Miaomiao Cai, Mengting Lin, Dan Li, Jiajia Li

**Affiliations:** 1 Department of Endocrinology II, Respiratory and Critical Care Medicine Ward III, The Third Affiliated Hospital of Wenzhou Medical University, Wenzhou, Zhejiang, China; 2 Respiratory and Critical Care Medicine Ward I, The Third Affiliated Hospital of Wenzhou Medical University, Wenzhou, Zhejiang, China; 3 Emergency and Critical Care Center, The Third Affiliated Hospital of Wenzhou Medical University, Wenzhou, Zhejiang, China

**Keywords:** chronic obstructive pulmonary disease, individual and family self-management theory, moderate-intensity exercise training, pulmonary rehabilitation, stable phase, supervised nursing

## Abstract

**Objective:**

To investigate the effects of supervised nursing combined with moderate-intensity exercise training based on the Individual and Family Self-Management Theory (IFSMT) on pulmonary rehabilitation in patients with stable chronic obstructive pulmonary disease (COPD), and to provide evidence for improving self-management ability, pulmonary function, and quality of life.

**Methods:**

Using a convenience sampling method, 112 patients with stable COPD admitted to a hospital in Zhejiang Province, China, were enrolled and randomly assigned to a control group or an intervention group using a random number table, with 56 patients in each group. The control group received routine nursing care for stable COPD, including disease education, medication guidance, and general rehabilitation advice. The intervention group received a comprehensive intervention consisting of supervised nursing combined with moderate-intensity exercise training based on the IFSMT framework. The intervention was implemented across three dimensions—context, process, and outcomes—focusing on enhancement of knowledge and health beliefs and family support integration (context), self-regulation guidance, individualized rehabilitation plan development, moderate-intensity exercise training, and continuous assessment and follow-up (process), as well as social facilitation and dynamic adjustment of the intervention program (outcomes). Before and after the intervention, the Chinese version of the Partners in Health scale (PIH), the Chinese-modified Fatigue Impact Scale (FIS), Borg dyspnea scale, and the 6-min walk distance (6MWD) were used to quantitatively assess and compare self-management ability, fatigue severity, pulmonary function, and exercise capacity between the two groups.

**Results:**

After systematic data collection, data management, and statistical analysis, the results showed that post-intervention forced expiratory volume in one second (FEV_1_) was higher in the intervention group than in the control group [1.05 (0.87, 1.40) vs. 0.89 (0.66, 1.17), Z = −2.91, *p* = 0.004]. Forced vital capacity (FVC) after the intervention was also significantly higher in the intervention group than in the control group (2.50 ± 0.63 vs. 1.91 ± 0.61, t = −5.06, *p* < 0.001). In terms of exercise capacity, the 6-min walk distance (6MWD) was significantly greater in the intervention group than in the control group (306.37 ± 91.98 m vs. 257.95 ± 92.08 m, t = −2.78, *p* = 0.006). Peak expiratory flow (PEF) after the intervention was also higher in the intervention group [4.31 (3.73, 5.99) vs. 2.24 (1.88, 2.92), Z = −7.63, *p* < 0.001]. Regarding symptom-related outcomes, Borg dyspnea scores were lower in the intervention group than in the control group [2.00 (1.00, 3.25) vs. 4.50 (3.00, 6.00), Z = −6.18, *p* < 0.001]. COPD Assessment Test (CAT) scores were also significantly lower in the intervention group [12.00 (10.00, 15.00) vs. 20.50 (16.75, 25.00), Z = −7.63, *p* < 0.001]. For fatigue and self-management outcomes, Fatigue Impact Scale (FIS) scores were significantly lower in the intervention group than in the control group [23.00 (20.00, 28.25) vs. 42.50 (32.25, 56.25), Z = −7.04, *p* < 0.001]. Similarly, Partners in Health (PIH) scores were lower in the intervention group [29.00 (22.00, 34.00) vs. 37.00 (28.00, 44.50), Z = −3.50, *p* < 0.001], indicating better self-management ability. In addition, Activities of Daily Living (ADL) scores were significantly lower in the intervention group than in the control group [23.00 (19.75, 25.25) vs. 32.50 (28.00, 38.00), Z = −7.87, *p* < 0.001], suggesting better functional status. Regarding modified Medical Research Council (mMRC) dyspnea grades after the intervention, in the intervention group, 5.36% (3/56) of patients were classified as grade 0, 57.14% (32/56) as grade 1, 32.14% (18/56) as grade 2, 1.79% (1/56) as grade 3, and 3.57% (2/56) as grade 4. In the control group, 1.79% (1/56) were classified as grade 0, 1.79% (1/56) as grade 1, 53.57% (30/56) as grade 2, 42.86% (24/56) as grade 3, and 0.00% (0/56) as grade 4. Overall, dyspnea severity was significantly lower in the intervention group than in the control group, and the between-group difference was statistically significant (*p* < 0.001).

**Conclusion:**

The comprehensive intervention integrating supervised nursing and moderate-intensity exercise training based on the Individual and Family Self-Management Theory (IFSMT) may improve self-management ability, reduce disease-related fatigue, enhance pulmonary function and activities of daily living, and improve quality of life in patients with stable COPD. These findings may provide a reference for pulmonary rehabilitation management in this population.

## Introduction

With the accelerating global population aging process and the combined effects of environmental factors, chronic respiratory diseases have become a major public health threat to human health. Among them, chronic obstructive pulmonary disease (COPD), characterized by a “triple-high” burden of high prevalence, high disability rate, and high mortality, has become the third leading cause of death worldwide, following ischemic heart disease and stroke (1). Epidemiological data indicate that approximately 545 million people worldwide suffer from chronic respiratory diseases. In China, the prevalence of COPD among individuals aged ≥20 years is 8.6%, rising to 13.7% among those aged ≥40 years, with nearly 100 million affected individuals and approximately one million deaths annually (2). As a progressive airflow-limiting disease, COPD presents with insidious symptoms in the early stage and is therefore easily overlooked. In the advanced stage, it can involve multiple organ systems and lead to severe complications such as respiratory failure and chronic pulmonary heart disease. Owing to the lack of a curative treatment, patients require lifelong therapy and management, imposing a substantial health and economic burden on individuals, families, and society (3).

In the early stage, the symptoms of COPD are usually mild, and patients often seek treatment only for common cough. As the disease progresses to advanced stages, it may affect multiple organ systems and lead to complications such as respiratory failure, spontaneous pneumothorax, chronic pulmonary heart disease, metabolic syndrome, and diabetes. Because COPD cannot be completely cured, patients require lifelong treatment and management. Standardized treatment can, to a certain extent, delay disease progression and help the disease enter a stable phase (4, 5). Stable COPD refers to the stage in which there are no acute exacerbations or worsening symptoms. Effective management during this stage can help delay disease progression, improve patients’ quality of life, and optimize long-term prognosis. As a critical period for disease management, the primary goals during the stable phase are to relieve clinical symptoms, enhance exercise tolerance, improve quality of life, and reduce the risk of acute exacerbations (5). Although a treatment system centered on the standardized use of inhaled medications has been established in clinical practice, patients in the stable phase largely rely on self-management or family-based care. Consequently, problems such as insufficient disease awareness, poor self-management ability, low medication adherence, and improper inhalation techniques are common (6). Previous studies have shown that treatment adherence is directly associated with COPD-related mortality, while factors such as perceived poor drug efficacy and concerns about adverse drug effects further exacerbate management difficulties (7). At the same time, most older adults patients with COPD experience functional decline, memory impairment, and low educational levels. In addition, disease-related anxiety, depression, and other negative emotions seriously impair their self-management capacity (8, 9). Although case management and integrated hospital–community–family management models have been applied in clinical practice, challenges such as high rates of acute exacerbations and unsatisfactory rehabilitation outcomes remain. Therefore, more targeted and innovative intervention strategies are urgently needed (10, 11).

The Individual and Family Self-Management Theory (IFSMT) explains the formation mechanisms of self-management behaviors in chronic diseases from three dimensions: context, process, and outcomes. The theory focuses on risk factor identification, integration of family resources, and the development of individual self-regulation, providing a well-established theoretical framework for behavioral interventions in chronic disease management (12). This theory has been shown to improve treatment adherence and prognosis in chronic disease management, including diabetes and gestational hypertension (13, 14). Supervised nursing is an intervention method that combines supervision and guidance in clinical nursing, with the core focus on overseeing the implementation of medical orders and nursing plans, as well as guiding clinical nursing practice (15). It balances patient guidance and medical collaboration, effectively improving rehabilitation adherence in patients from different backgrounds (16). Exercise training is the core and foundation of pulmonary rehabilitation for COPD patients, as patients often experience exercise fatigue due to long-term illness. The primary reason for exercise limitation is the decline in cardiopulmonary function, and exercise intensity is a key factor affecting rehabilitation outcomes (17). Moderate-intensity training has been confirmed to balance efficacy and safety, enhancing muscle strength, improving cardiopulmonary function, and alleviating dyspnea, with superior outcomes compared to high-intensity interval training and basic healthcare exercises (18).

A review of the existing literature shows that current studies mostly apply the IFSMT theory, supervised nursing, or exercise training individually. This results in single-dimensional interventions, lack of theoretical guidance, and difficulty in maintaining long-term rehabilitation behaviors. The application of a systematically integrated model combining all three in pulmonary rehabilitation for stable COPD has not yet been reported, which is the research question and innovation of this study. Based on this, this study is guided by IFSMT theory, integrating the advantages of supervised nursing’s full-course supervision and the physiological improvement value of moderate-intensity exercise training. The study constructs a unified intervention model and designs a randomized controlled trial to clarify the effects of this model on self-management ability, lung function, exercise endurance, and quality of life in patients with stable COPD. This aims to provide evidence for optimizing the pulmonary rehabilitation plan for COPD patients in the stable phase. The study is reported as follows.

## Materials and methods

### Participants

#### Study design

This study was a single-center, parallel-group, randomized controlled trial, with a 1:1 allocation ratio between the intervention group and the control group. The study was conducted from July 2025 to January 2026 in the Department of Respiratory Medicine of a tertiary hospital in Zhejiang Province, China. The trial was registered in the Chinese Clinical Trial Registry (registration no. ChiCTR2500103664) and was conducted in accordance with the Consolidated Standards of Reporting Trials (CONSORT) guidelines and the Declaration of Helsinki.

#### Setting and participant recruitment

This study was conducted in the Department of Respiratory Medicine of a hospital in Rui’an, Zhejiang Province, China. Participants were recruited from both the outpatient clinic and inpatient ward of the Department of Respiratory Medicine. All enrolled patients were strictly defined as being in the stable phase of COPD, with no acute exacerbations or newly developed respiratory infections within the previous 3 months, and their condition remained stable. Hospitalized patients were not admitted due to acute exacerbation of COPD, but rather for routine conditioning and regular inpatient evaluation. During outpatient consultations and ward rounds, the research team conducted preliminary screening according to the inclusion and exclusion criteria. Patients and their family members were fully informed about the study objectives, intervention protocol, data collection procedures, follow-up arrangements, and potential risks. Participants who volunteered to participate and signed written informed consent were enrolled in the study.

#### Eligibility criteria

##### Inclusion criteria

Patients were included if they met the diagnostic criteria for chronic obstructive pulmonary disease (COPD) according to the 2023 GOPD guidelines and were confirmed by pulmonary function testing (19), chest X-ray, and laboratory examinations; had stable clinical signs and dyspnea symptoms for ≥1 month; had normal mental and cognitive function with adequate ability to understand and complete the questionnaires; were aged ≥40 years; and voluntarily participated in the study with written informed consent.

##### Exclusion criteria

Patients were excluded if they had comorbid bronchial asthma, pulmonary interstitial fibrosis, or other diseases that could affect the assessment of pulmonary function; joint or muscular disorders that could impair motor function; cerebrovascular or cardiovascular diseases such as stroke or myocardial infarction; major organ dysfunction or severe systemic diseases; a confirmed diagnosis of malignant tumors; severe complications such as chronic pulmonary heart disease, rupture of emphysema, or rupture of pulmonary bullae; or were unable to complete the intervention and follow-up procedures.

#### Randomization and allocation

To minimize selection bias and ensure balanced group assignment, randomization was conducted using a random number table. Eligible patients with stable COPD were consecutively enrolled via convenience sampling from both the outpatient clinic and inpatient ward and sequentially numbered from 1 to 120. An independent researcher, who did not participate in intervention implementation or data collection, generated corresponding random numbers using the random number table. Participants were then assigned to the intervention or control group based on the odd or even value of their assigned random number. The group allocation sequence was prepared, sealed, and stored by a designated person throughout the study. Recruitment staff, clinical nurses, and outcome assessors were blinded to the allocation in advance. Allocation concealment was strictly maintained to effectively control selection and implementation bias.

#### Blinding

Because the intervention involved supervised nursing and exercise training with clearly defined procedures, blinding of the participants and the intervention providers was not feasible. However, the personnel responsible for group assignment, data collection, and statistical analysis were blinded to group allocation in order to minimize information bias.

#### Sample size calculation

This study was designed as an experimental study. The sample size was estimated using the formula for comparing the means of two independent samples:

N1 = N2 = 2[(tα_/2_ + t_β/2_)S/*δ*], where N₁ and N₂ represent the sample sizes of the two groups. In this study, the allocation ratio between the two groups was set at 1:1. Based on previous pulmonary rehabilitation studies involving patients with stable COPD (6, 8), the parameters were set as S = 8.8, *δ* = 5.6, *α* = 0.05, and *β* = 0.10. According to statistical tables, tα_/2_ = 1.96 and t_β/2_ = 1.282. Substituting these parameters into the formula indicated that approximately 49 participants were required in each group. Considering potential sampling error and loss to follow-up during the study, the sample size was increased by 10%, resulting in a minimum required sample size of 54 participants per group.

### Interventions methods

#### Control group: usual care

The control group received routine nursing care for patients with stable COPD. The specific measures included the distribution of health education pamphlets after admission and the provision of disease-related education, covering the etiology, symptoms, treatment principles, and prognosis of COPD. Medication guidance was provided in accordance with medical prescriptions, with detailed explanations of the correct use of inhaled medications, including administration methods, dosage, timing, and precautions; operational demonstrations were given when necessary. Patients were instructed in effective coughing and sputum expectoration techniques, and the importance of smoking cessation and alcohol abstinence was emphasized, along with avoiding exposure to harmful substances such as dust and smoke. The indications and precautions for long-term oxygen therapy were explained, and patients were assisted in establishing a regular daily routine. Dietary guidance was provided, advising patients to avoid spicy and greasy foods, to increase water intake, and to consume more fruits and vegetables. Patients were also encouraged to choose appropriate functional exercises according to their individual conditions, such as walking, slow walking, and breathing exercises; however, no unified exercise intensity or frequency was prescribed. One telephone follow-up was conducted 3 months after discharge.

#### Intervention group: supervised nursing combined with moderate-intensity exercise training based on IFSMT

The intervention group received a comprehensive intervention program for 3 months after discharge. Guided by the Individual and Family Self-Management Theory (IFSMT), this program integrated supervised nursing with moderate-intensity exercise training. The specific implementation is described as follows.

##### Establishment of the intervention team

An IFSMT-based supervised nursing intervention team was established, consisting of one attending physician from the Department of Respiratory Medicine, one head nurse, one rehabilitation therapist, and 8–10 clinical nurses with more than 5 years of experience in respiratory nursing. The attending physician was responsible for disease assessment and review of the intervention protocol; the head nurse was responsible for overall coordination; the rehabilitation therapist was responsible for developing the exercise training program; and the clinical nurses were responsible for the specific implementation of the intervention and follow-up supervision.

##### Individual assessment and construction of the theoretical framework

Upon admission, clinical nurses conducted one-to-one interviews with patients to collect baseline information, including sex, age, disease duration, smoking history, comorbidities, and COPD pulmonary function classification. Meanwhile, patients’ disease progression, discomfort-related complaints, level of disease awareness, and current status of self-management were assessed. Based on these assessments, key intervention priorities were identified across the three dimensions of context, process, and outcomes. The details of the intervention framework are presented in Table 1.

**Table 1:** Intervention program.

Dimension	Core component	Specific implementation	Timing	Implementer
Context intervention	Enhancement of knowledge and health beliefs	1. A semi-structured interview (15–20 min per patient) was conducted using simplified questions tailored to the patient’s disease course and educational level, focusing on key misconceptions regarding disease understanding, medication necessity, value of rehabilitation training, avoidance of environmental triggers, and early warning signs of recurrence. Patients’ questions were documented. 2. Based on the interview, a personalized illustrated educational booklet was developed for health education, emphasizing standardized medication use, correcting the misconception of stopping medication after symptom relief, explaining the gradual progression of rehabilitation training to avoid over- or under-training, and clarifying specific protective measures for home and outdoor settings.	The first interview and Q&A session was completed within 24 h of admission. After discharge, online follow-up interviews were conducted once every two weeks. For patients who did not meet the reassessment criteria, additional education was provided within 48 h through video consultation or home visits, with reinforcement of relevant health information delivered simultaneously via WeChat.	Clinical nurses
	Family support integration	Patients were grouped according to age and disease severity, with 5–7 patients per group to facilitate mutual support. Each group was assigned one supervising nurse, and a WeChat group was established. 1. Family collaboration: a family collaboration workflow was developed, including patients’ daily self-checks of medication use and exercise records. Family members were responsible for medication reminders at 7:00 a.m. and accompanying patients during exercise after dinner. a daily record form with dual signatures from both patients and family members was provided and signed by family members upon completion each day. 2. WeChatgroup management: health education messages (e.g., daily dietary tips) were sent at 8:00 a.m., and patient rehabilitation check-in cases were shared at 8:00 p.m. 3. Online q&a sessions: online q&a sessions were conducted every Wednesday at 7:00 p.m.; questions were collected in advance, physicians were invited to provide responses, and the q&a records were compiled and redistributed. 4. Online consultation: an AI-based follow-up system from the hospital was introduced in the WeChat group to answer simple questions. In addition, a live consultation session was conducted every Wednesday at 7:00 p.m. patients’ questions were collected in advance, and physicians were invited to provide real-time online responses. The consultation records were organized into documents and redistributed to participants afterward.	Within 1 week after admission, grouping and educational sessions were completed; the WeChat groups were maintained until 3 months after discharge.	Supervising nurses
Process intervention	Self-regulation guidance	1. Identification of key problems using a problem checklist: nurses distributed a self-management problem checklist covering eight core domains: medication adherence, exercise performance, smoking and alcohol cessation, dietary control, daily routine regularity, emotional status, environmental protection, and symptom monitoring. Patients were guided to identify items they found difficult and to rate the impact of each problem on rehabilitation using a 1–10 scale (10 indicating the greatest impact). 2. Nurse-assisted prioritization and goal setting: nurses reviewed the checklist jointly with patients and family members, supplemented problems based on clinical observation, and applied a combined scoring and clinical-priority approach to rank issues. The top three key problems were identified, and self-regulation goals targeting these problems were established. Goals were adjusted weekly, and after discharge, guidance was delivered online via WeChat every Monday.	Problem identification was completed within 1 week after admission; during hospitalization and for 3 months after discharge, adjustments were made weekly.	Clinical nurses + patients + family members
	Individualized rehabilitation plan development – medication management	1. Clinical nurses provided short instructional videos and on-site demonstrations on the correct use of inhaled medications and rehabilitation exercises. Operational videos were recorded for review. 2. Family members received concurrent training, and a medication record form was provided to document daily medication timing, dosage, and adverse reactions. 3. Supervising nurses reviewed the medication records weekly to ensure medication adherence rates of ≥95%.	Training was completed within 72 h after admission; daily records were kept during hospitalization and for 3 months after discharge, with weekly checks of completion.	Supervising nurses + family members
	Lifestyle management	1. A smoking and alcohol cessation schedule was developed, with daily recording by family members. 2. A customized light-diet plan was provided, and a dietary record form was issued to document daily salt and oil intake and types of food consumed. 3. A daily routine record form was used, encouraging bedtime before 22:00 and a minimum sleep duration of ≥7 h per night.	Implemented continuously during hospitalization and for 3 months after discharge, with weekly follow-up checks.	Supervising nurses + patients + family members
	Emotional regulation	1. Peer education at admission: a 30-min peer education session was conducted at admission, using rehabilitation cases to enhance patient confidence, relieve anxiety, and support emotional regulation. 2. Case-based education: online case-based education sessions featuring patients with good adherence were organized in the second and sixth weeks after discharge to enhance patients’ confidence in disease management.	Assessments were conducted at admission and at the 2nd and 6th weeks after discharge.	Clinical nurses + patients
	Moderate-intensity exercise training	1. A rehabilitation therapist assessed baseline exercise capacity and prescribed an individualized program at 55–75% of maximum heart rate. 2. Lower-limb aerobic exercise (slow walking, jogging, or swimming) was performed for 30 min per session at 4–6 km/h, three times per week. 3. Resistance band breathing exercise training the training consisted of three movements. ① Seated rowing: the patient sat upright with the resistance band looped around the soles of the feet and both hands holding the band. The arms were extended during inhalation and pulled toward the abdomen during exhalation. ② Overhead shoulder press: the patient sat upright while holding the resistance band and raised both arms above the head. The arms were opened laterally during inhalation and lifted upward during exhalation before returning to the starting position. ③ Breathing stretch: the patient stood upright while holding the resistance band in front of the chest. During inhalation, the band was pulled laterally to expand the chest, followed by relaxation during exhalation. ④ Duration: each movement was performed 10–15 repetitions per set for 3–5 sets. Training lasted 10 min per day during weeks 1–6 after discharge, and twice daily for 20 min per session during weeks 7–12. 4. Abdominal sandbag breathing exercise training. Patients used a 1–3 kg clean sandbag and wore loose clothing while performing the training in a quiet and well-ventilated environment. Patients lay in a supine position with knees flexed and feet flat, with a thin pillow placed under the head if necessary. The abdomen was kept relaxed. The sandbag was placed on the upper abdomen without compressing the chest. during the exercise, patients inhaled through the nose while allowing the abdomen to rise and lift the sandbag, maintaining this position for 1–2 s. the abdominal muscles were then contracted to raise the sandbag 5–10 cm, held for 3–5 s, followed by slow exhalation as the sandbag returned to the resting position. The exercise was performed 10–15 repetitions per set for 3–5 sets per day. Training lasted 10 min per day during weeks 1–6 after discharge, and twice daily for 20 min per session during weeks 7–12, with gradual progression. 5. family-assisted exercise monitoring family members accompanied patients during exercise and recorded activity in an exercise log. Weekly family follow-up assessments were conducted to evaluate adherence, with a target exercise completion rate of ≥90%.	Implemented continuously during hospitalization and for 3 months after discharge, 1–2 times daily, with weekly follow-up assessments.	Rehabilitation therapist + supervising nurses + patients + family members
	Assessment and follow-up	1. In-hospital supervision: supervising nurses conducted daily checks of rehabilitation adherence during hospitalization. 2. Pre-discharge planning: one day before discharge, a home-based plan was developed with patients and family members. 3. Within 3 months after discharge: patients received one telephone follow-up and one home visit per month to evaluate adherence to the intervention and document the findings in a follow-up record form. For patients with more severe conditions or poor self-management ability, the follow-up frequency was increased to once per week. These follow-ups mainly involved on-site assessment of the home environment and rehabilitation training status, and individualized nursing support was provided accordingly.	Daily communication during hospitalization; after discharge, one monthly telephone follow-up plus one home visit per week.	Supervising nurses
Outcome intervention	Social facilitation	1. A 30-min family discussion meeting was conducted before discharge to clarify patient self-management boundaries and the scope of family support. 2. After discharge, family members were informed that patients should participate in two community activities if their clinical condition permitted. 3. For patients with good adherence, the frequency of family reminders was reduced to once every two days or less.	The discussion meeting was completed 3 days before discharge.	Supervising nurses + patients + family members
	Dynamic adjustment of the intervention program	1. Exercise plans were adjusted weekly based on exercise logs and symptom records. 2. At 3 months after discharge, comprehensive reassessment (pih scale, fatigue scale, borg score, and 6mwd) was conducted to inform program adjustment. 3. In case of adverse events, the program was adjusted immediately and follow-up was conducted.	Adjusted weekly during hospitalization and for 3 months after discharge.	Rehabilitation therapist + supervising nurses + attending physician

### Quality control

Before implementation, the intervention protocol was developed based on a comprehensive review of domestic and international literature and official guidelines, and was refined through expert panel discussions to ensure scientific rigor and feasibility. Prior to study initiation, all members of the intervention team received standardized training covering IFSMT principles, supervised nursing skills, exercise training procedures, and the use of assessment scales; only those who passed the competency assessment were allowed to participate. All questionnaires were administered using standardized instructions. Trained researchers assisted participants in completing the questionnaires, and interviewer-administered completion was adopted for participants with lower educational levels to ensure accuracy. Questionnaires were collected and checked on site, and invalid questionnaires were excluded. Data entry was performed independently by two researchers, followed by cross-checking to ensure data accuracy. During the intervention period, regular audits and reviews of follow-up records were conducted to monitor protocol adherence, and deviations were promptly identified and corrected.

Given the 3-month follow-up period of this study, a series of adherence management measures were implemented to ensure patient compliance with the intervention. During the first 3 months after discharge, patients received one telephone follow-up and one home visit per month. Medication records and exercise logs were reviewed weekly to ensure a medication adherence rate of ≥95% and an exercise completion rate of ≥90%. In addition, a family-assisted supervision mechanism was established, whereby family members were responsible for reminding, accompanying, and recording the patients’ activities, with confirmation through dual signatures. A WeChat group was also used for daily check-ins, online education, and consultation to enhance patients’ rehabilitation motivation. For patients showing decreased adherence, additional intervention was provided within 48 h through video consultation or home visits. These measures helped ensure standardized implementation of the intervention and maintained the quality of the study. Adverse events were monitored throughout the study. No intervention-related adverse events were reported during the entire intervention period.

### Outcome measures

All participants in both groups completed the following assessments at baseline (within 48 h after admission) and at 3 months after the intervention. All data were recorded using standardized data collection forms to ensure completeness and consistency.

#### General characteristics

Baseline demographic and clinical characteristics were collected, including demographic variables (age, sex, height, weight, educational level, occupation, and marital status), socioeconomic information, lifestyle factors (smoking status, smoking duration, and alcohol consumption), and clinical characteristics (comorbidities, duration since COPD diagnosis, COPD severity classification, number of previous hospitalizations for COPD, medication use, medication adherence, and presence of a fixed caregiver).

#### Self-management ability

Self-management ability was assessed using the Chinese version of the Partners in Health (PIH) scale (20). The scale comprises 12 items across three domains: knowledge (7 items), coping (3 items), and adherence (2 items). Each item is scored on a 0–8 scale (0 = very satisfied, 8 = very dissatisfied), yielding a total score ranging from 0 to 96. Lower scores indicate better self-management ability. The Cronbach’s *α* coefficient of this scale was 0.890, indicating good reliability and validity.

#### Disease-related fatigue

Disease-related fatigue was assessed using the Chinese revised version of the Fatigue Impact Scale (FIS) (21). The scale consists of 25 items across four domains: physical functioning (7 items), cognitive functioning (6 items), psychosocial functioning (6 items), and behavioral functioning (6 items). Each item is scored on a 0–4 scale (0 = no problem, 4 = severe problem), yielding a total score ranging from 0 to 100. Higher scores indicate greater fatigue severity.

#### Pulmonary function–related outcomes

##### Objective pulmonary function parameters

Objective pulmonary function parameters were measured using a spirometer, including forced expiratory volume in 1 s (FEV₁, L), forced vital capacity (FVC, L), the FEV₁/FVC ratio, and peak expiratory flow (PEF, L/s). The FEV₁/FVC ratio is a key indicator for the diagnosis of COPD, with a value <70% indicating airflow limitation.

##### Dyspnea assessment

The modified Medical Research Council (mMRC) dyspnea scale was used to assess dyspnea severity using a 0–4 grading system: grade 0 = no dyspnea; grade 1 = dyspnea when walking quickly; grade 2 = walking slower than people of the same age on level ground or needing to stop for rest; grade 3 = needing to stop for rest after walking within 100 meters or after a few minutes on level ground; and grade 4 = dyspnea at rest (22).

The Borg dyspnea scale was also used, with scores ranging from 0 to 10 (0 = no dyspnea, 10 = maximal dyspnea), to assess the severity of dyspnea at rest (23).

##### Exercise capacity assessment

Exercise capacity was assessed using the 6-min walk distance (6MWD) test (24). The total distance walked (in meters) during a 6-min period was recorded. The test was conducted in a corridor longer than 30 m, and patients were instructed to walk as fast as possible for 6 min.

#### Disease impact assessment

Disease impact was assessed using the Chronic Obstructive Pulmonary Disease Assessment Test (CAT) (25). The CAT consists of eight items covering cough, sputum production, chest tightness, dyspnea, sleep disturbance, and energy levels. Each item is scored from 0 to 5, yielding a total score ranging from 0 to 40, with higher scores indicating a greater impact of COPD on daily life.

#### Activities of daily living

Activities of daily living were assessed using the Activities of Daily Living (ADL) scale, developed by Graf (26) and Zhao et al. (27). The scale comprises two subscales: basic activities of daily living (6 items) and instrumental activities of daily living (8 items). Each item is rated on a 1–4 scale (able to perform independently, some difficulty, requiring assistance, unable to perform), with higher scores indicating poorer functional ability.

### Statistical analysis

Statistical analyses were performed using SPSS version 26.0. Continuous variables were first assessed for normality using the Shapiro–Wilk test and for homogeneity of variance using Levene’s test. Data with a normal distribution and homogeneous variance were expressed as mean ± standard deviation (SD), and between-group comparisons were conducted using independent-samples *t* tests, while within-group pre- and post-intervention comparisons were performed using paired *t* tests. Data that did not meet assumptions of normality or homogeneity of variance were expressed as median (interquartile range) [M (IQR)], and between-group comparisons were performed using the Wilcoxon rank-sum test. Categorical variables were expressed as number (percentage) [*n* (%)], and comparisons between groups were conducted using the chi-square test. A two-sided *p* value < 0.05 was considered statistically significant.

## Results

### Baseline characteristics of the study population

A total of 120 patients were assessed for eligibility. Of these, five patients declined participation and were excluded. Finally, 115 patients were enrolled and randomly allocated to the control group (*n* = 57) or the intervention group (*n* = 58). The control group received routine nursing care during the stable phase of COPD, while the intervention group received a comprehensive intervention consisting of supervised nursing combined with moderate-intensity exercise training based on the IFSMT framework.

**Figure 1:**
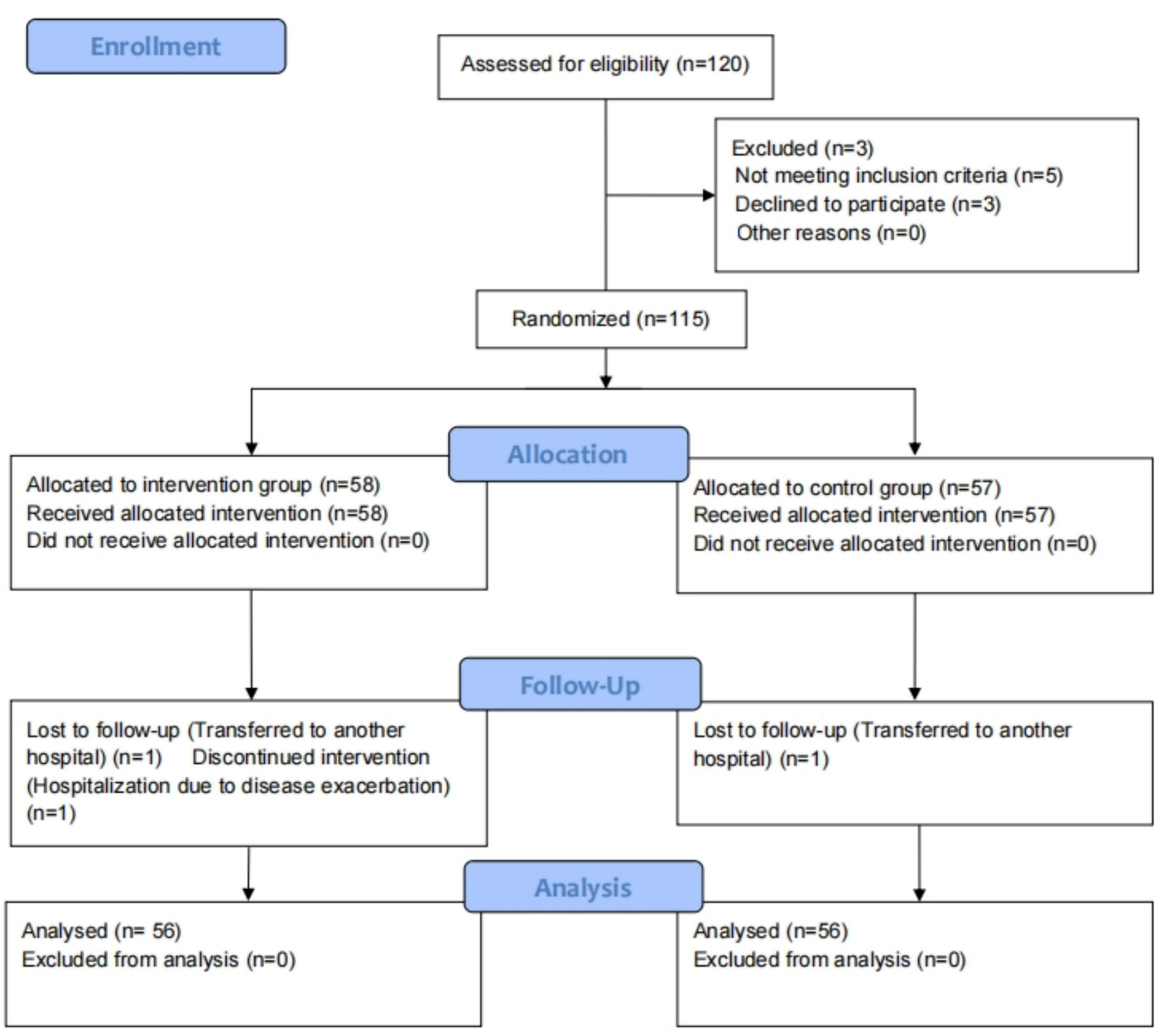
CONSORT flow diagram.

During the study period, one patient in the control group was lost to follow-up due to transfer to another hospital, and two patients in the intervention group were lost to follow-up (one due to transfer and one due to disease exacerbation requiring hospitalization). Ultimately, 56 patients in each group completed the final assessment (Figure 1).

There were no statistically significant differences between the two groups in terms of age, height, weight, smoking duration, sex, educational level, occupation, marital status, smoking status, alcohol consumption, COPD severity classification, medication adherence, presence of a fixed caregiver, comorbidities, or medication use. In addition, baseline measures—including fatigue severity, self-management ability, activities of daily living (ADL), forced expiratory volume in 1 s (FEV₁), forced vital capacity (FVC), FEV₁/FVC ratio, peak expiratory flow (PEF), Borg dyspnea score, COPD Assessment Test (CAT) score, mMRC dyspnea grade, and 6-min walk distance (6MWD)—did not differ significantly between groups (all *p* > 0.05), indicating good comparability. Detailed baseline characteristics are presented in Table 2.

**Table 2:** Baseline characteristics of participants in the control and intervention groups.

Variable	*N* = 112	Control group (*n* = 56)	Intervention group (*n* = 56)	Statistic	*p*
Age	73.05 ± 9.70	74.61 ± 10.15	71.50 ± 9.05	t = 1.71	0.090
Height	163.43 ± 7.45	163.56 ± 8.11	163.30 ± 6.80	t = 0.18	0.855
Weight	56.50 (50.00, 65.00)	55.00 (50.75, 62.38)	57.25 (50.00, 66.62)	Z = −0.92	0.360
Smoking duration (years)	26.00 (0.00, 39.00)	27.00 (0.00, 39.25)	20.00 (0.00, 38.00)	Z = −0.22	0.823
Sex				χ^2^ = 0.29	0.589
Male	96 (85.71)	47 (83.93)	49 (87.50)		
Female	16 (14.29)	9 (16.07)	7 (12.50)		
Educational level				-	1.000
Junior high school or below	110 (98.21)	55 (98.21)	55 (98.21)		
Senior high school or vocational school	1 (0.89)	1 (1.79)	0 (0.00)		
College degree or above	1 (0.89)	0 (0.00)	1 (1.79)		
Occupation				-	0.789
Manual workers	16 (14.29)	7 (12.50)	9 (16.07)		
Mental workers	2 (1.79)	1 (1.79)	1 (1.79)		
Service industry workers	5 (4.46)	4 (7.14)	1 (1.79)		
Self-employed	16 (14.29)	8 (14.29)	8 (14.29)		
Retired or unemployed	73 (65.18)	36 (64.29)	37 (66.07)		
Marital status				-	0.709
Married	90 (80.36)	44 (78.57)	46 (82.14)		
Unmarried	3 (2.68)	1 (1.79)	2 (3.57)		
Divorced or widowed	19 (16.96)	11 (19.64)	8 (14.29)		
Smoking				χ^2^ = 0.40	0.526
Yes	81 (72.32)	39 (69.64)	42 (75.00)		
No	31 (27.68)	17 (30.36)	14 (25.00)		
Alcohol consumption				χ^2^ = 3.66	0.161
Long-term heavy drinking	12 (10.71)	7 (12.50)	5 (8.93)		
Occasional drinking	31 (27.68)	11 (19.64)	20 (35.71)		
No alcohol consumption	69 (61.61)	38 (67.86)	31 (55.36)		
COPD severity classification				-	0.489
Grade I	1 (0.89)	0 (0.00)	1 (1.79)		
Grade II	31 (27.68)	13 (23.30)	18 (32.10)		
Grade III	52 (46.43)	29 (51.70)	23 (41.10)		
Grade IV	28 (25.00)	14 (25.00)	14 (25.00)		
Medication adherence				χ^2^ = 0.06	0.809
Good	91 (81.25)	46 (82.14)	45 (80.36)		
Poor	21 (18.75)	10 (17.86)	11 (19.64)		
Presence of a fixed caregiver				χ^2^ = 0.04	0.843
Yes	73 (65.18)	36 (64.29)	37 (66.07)		
No	39 (34.82)	20 (35.71)	19 (33.93)		
Comorbidities				-	0.678
None	28 (25.00)	12 (21.43)	16 (28.57)		
Cardiovascular diseases	39 (34.82)	22 (39.29)	17 (30.36)		
Diabetes mellitus	5 (4.46)	3 (5.36)	2 (3.57)		
Osteoporosis	1 (0.89)	1 (1.79)	0 (0.00)		
Two or more comorbidities	39 (34.82)	18 (32.14)	21 (37.50)		
Medication use				-	0.161
Bronchodilators	3 (2.68)	3 (5.36)	0 (0.00)		
Glucocorticoids	3 (2.68)	3 (5.36)	0 (0.00)		
Expectorants	2 (1.79)	2 (3.57)	0 (0.00)		
Antibiotics	4 (3.57)	2 (3.57)	2 (3.57)		
Phosphodiesterase inhibitors	3 (2.68)	1 (1.79)	2 (3.57)		
Enzyme inhibitors and antioxidants	2 (1.79)	1 (1.79)	1 (1.79)		
Combination therapy	95 (84.82)	44 (78.57)	51 (91.07)		
Fatigue Impact Scale (FIS) score at admission	54.24 ± 12.12	55.54 ± 13.55	52.95 ± 10.48	t = 1.13	0.260
Partners in Health (PIH) scale score at admission	50.13 ± 11.68	51.86 ± 12.61	48.41 ± 10.50	t = 1.57	0.119
Forced expiratory volume in 1 s (FEV₁) at admission	0.90 (0.69, 1.23)	0.88 (0.65, 1.15)	0.91 (0.73, 1.27)	Z = −1.28	0.200
Forced vital capacity (FVC) at admission	1.87 (1.47, 2.23)	1.75 (1.27, 2.18)	1.92 (1.51, 2.26)	Z = −1.27	0.204
Peak expiratory flow (PEF) at admission	2.01 (1.55, 2.92)	2.01 (1.65, 2.59)	2.03 (1.35, 3.22)	Z = −0.07	0.944
Borg dyspnea score at admission	5.00 (4.00, 6.00)	5.00 (4.00, 6.00)	5.00 (4.00, 6.00)	Z = −0.07	0.945
6-min walk distance (6MWD) at admission	213.50 (158.00, 286.50)	205.50 (151.75, 284.50)	217.00 (172.50, 291.50)	Z = −0.93	0.355
COPD Assessment Test (CAT) score at admission	27.00 (24.00, 32.00)	27.00 (23.75, 32.25)	27.00 (24.00, 32.00)	Z = −0.26	0.793
Activities of Daily Living (ADL) score at admission	40.50 (37.00, 48.00)	41.50 (37.00, 48.00)	40.00 (37.00, 48.25)	Z = −0.01	0.993
mMRC dyspnea grade at admission				-	0.398
Grade 1	0 (0.00)	0 (0.00)	0 (0.00)		
Grade 1	1 (0.89)	1 (1.79)	0 (0.00)		
Grade 3	5 (4.46)	1 (1.79)	4 (7.14)		
Grade 4	62 (55.36)	33 (58.93)	29 (51.79)		

### Comparison of outcome measures between the two groups

After the intervention, the intervention group demonstrated significantly higher forced expiratory volume in 1 s (FEV₁), forced vital capacity (FVC), 6-min walk distance (6MWD), FEV₁/FVC ratio, and peak expiratory flow (PEF) compared with the control group (*p* < 0.05). In contrast, the intervention group showed significantly lower Borg dyspnea scores, COPD Assessment Test (CAT) scores, Fatigue Impact Scale (FIS) scores, and Partners in Health (PIH) scale scores than the control group (*p* < 0.001). In addition, dyspnea severity assessed by the mMRC scale was significantly better in the intervention group than in the control group (*p* < 0.05). Detailed results are presented in Table 3.

**Table 3:** Comparison of outcome measures between the control and intervention groups.

Variable	*n* = 112	Control group (*n* = 56)	Intervention group (*n* = 56)	Statistic	*p*
Forced expiratory volume in 1 s (FEV₁) after intervention	0.97 (0.75, 1.27)	0.89 (0.66, 1.17)	1.05 (0.87, 1.40)	Z = −2.91	0.004
Forced vital capacity (FVC) after intervention	2.20 ± 0.68	1.91 ± 0.61	2.50 ± 0.63	t = −5.06	<0.001
6-min walk distance (6MWD) after intervention	282.16 ± 94.79	257.95 ± 92.08	306.37 ± 91.98	t = −2.78	0.006
Peak expiratory flow (PEF) after intervention	3.56 (2.24, 4.42)	2.24 (1.88, 2.92)	4.31 (3.73, 5.99)	Z = −7.63	<0.001
Borg dyspnea score after intervention	3.00 (2.00, 5.00)	4.50 (3.00, 6.00)	2.00 (1.00, 3.25)	Z = −6.18	<0.001
COPD Assessment Test (CAT) score after intervention	16.00 (12.00, 21.25)	20.50 (16.75, 25.00)	12.00 (10.00, 15.00)	Z = −7.63	<0.001
Fatigue Impact Scale (FIS) score after intervention	29.50 (22.00, 42.25)	42.50 (32.25, 56.25)	23.00 (20.00, 28.25)	Z = −7.04	<0.001
Partners in Health (PIH) scale score after intervention	31.50 (24.00, 40.00)	37.00 (28.00, 44.50)	29.00 (22.00, 34.00)	Z = −3.50	<0.001
Activities of Daily Living (ADL) score after intervention	27.00 (23.00, 32.25)	32.50 (28.00, 38.00)	23.00 (19.75, 25.25)	Z = −7.87	<0.001
mMRC dyspnea grade after intervention				-	<0.001
Grade 0	2 (1.79)	1 (1.79)	3 (5.36)		
Grade 1	4 (3.57)	1 (1.79)	32 (57.14)		
Grade 2	33 (29.46)	30 (53.57)	18 (32.14)		
Grade 3	48 (42.86)	24 (42.86)	1 (1.79)		
Grade 4	25 (22.32)	0 (0.00)	2 (3.57)		

## Discussion

### The IFSMT-based comprehensive intervention significantly improves self-management ability in patients with stable COPD

The results of this study showed that, after the intervention, the total score of the Chinese version of the Partners in Health (PIH) scale was lower in the intervention group than in the control group, indicating that the comprehensive intervention effectively enhanced patients’ self-management ability. Insufficient self-management among patients with stable COPD is primarily attributed to inadequate disease-related knowledge, weak self-regulation capacity, and insufficient family support (28). Guided by the IFSMT framework, the contextual intervention component of this study identified key cognitive misconceptions through semi-structured interviews and reinforced health beliefs and family collaboration through individualized education and joint family sessions, thereby addressing deficits in disease awareness at the source. Within the process intervention component, the use of a problem checklist facilitated the identification of key self-management challenges, enabling the formulation of phased goals and individualized rehabilitation plans. Combined with supervised nursing and weekly follow-up, this approach effectively improved medication adherence and exercise implementation. Previous studies have demonstrated that IFSMT-based interventions can enhance patients’ self-management engagement through a closed-loop model encompassing context, process, and outcomes (29). In addition, the continuous supervision and dynamic adjustment inherent in supervised nursing further ensured the feasibility and implementation fidelity of the intervention. The synergistic application of these strategies contributed to comprehensive improvements in self-management ability, which is consistent with the findings of the present study.

### The IFSMT-based comprehensive intervention effectively reduces disease-related fatigue in patients with stable COPD

After the intervention, the total score of the Chinese revised Fatigue Impact Scale was lower in the intervention group than in the control group, indicating that the comprehensive intervention effectively alleviated fatigue across physical, cognitive, psychological, and behavioral domains. Fatigue in patients with COPD is closely associated with impaired pulmonary function, reduced exercise tolerance, and increased psychological stress (30). In the present study, moderate-intensity exercise training, incorporating lower-limb aerobic exercise, resistance training, and respiratory muscle training, progressively enhanced cardiopulmonary function and muscular endurance, thereby reducing post-exercise fatigue. In addition, peer education embedded within the intervention effectively alleviated negative emotional states such as anxiety and depression, mitigating fatigue driven by psychological factors. Moreover, IFSMT emphasizes the critical role of family support; supervision and emotional support from family members can reduce patients’ feelings of loneliness and psychological burden, indirectly contributing to fatigue reduction (31). Nyberg et al. demonstrated that moderate-intensity exercise training can reduce exertional fatigue by improving muscular endurance (18). Building on these findings, the present study integrated peer education and family support with exercise-based interventions to establish a multidimensional fatigue management framework, which further enhanced fatigue relief.

### The IFSMT-based comprehensive intervention significantly improves pulmonary function and exercise capacity in patients with stable COPD

After the intervention, objective pulmonary function parameters (FEV₁, FVC, FEV₁/FVC ratio, and PEF), dyspnea measures (mMRC grade and Borg score), and exercise capacity (6MWD) were all better in the intervention group than in the control group, indicating that the comprehensive intervention effectively improved pulmonary function and physical performance in patients with stable COPD. Airway obstruction and respiratory muscle dysfunction are key pathological mechanisms underlying pulmonary function decline in COPD, and exercise training represents a core component of pulmonary rehabilitation (32). In this study, rehabilitation therapists developed individualized moderate-intensity exercise programs based on baseline pulmonary function and physical capacity, with exercise intensity maintained at 55–75% of maximum heart rate. This intensity range minimized dyspnea exacerbation associated with high-intensity exercise while providing sufficient physiological stimulation to enhance respiratory muscle strength and cardiopulmonary function. Previous studies have demonstrated that structured moderate-intensity exercise training significantly improves 6MWD and FEV₁ in patients with COPD (33, 34). By ensuring the standardization and continuity of exercise training through supervised nursing and integrating the comprehensive support framework of IFSMT, the present study achieved synergistic improvements in pulmonary function and exercise capacity.

### The IFSMT-based comprehensive intervention significantly reduces disease impact and improves activities of daily living in patients with stable COPD

After the intervention, the intervention group showed lower COPD Assessment Test (CAT) scores and better Activities of Daily Living (ADL) scores than the control group, indicating that the IFSMT-based comprehensive intervention improved patients’ overall living status by alleviating disease impact and enhancing functional independence. The reduction in CAT scores can be attributed to the closed-loop, multidimensional nature of the intervention. Standardized medication guidance reduced symptom frequency, lifestyle management lowered the risk of acute exacerbations, emotional regulation improved sleep quality, and moderate-intensity exercise training alleviated dyspnea, collectively reducing the interference of COPD with daily activities such as eating, sleeping, and social participation (35). Improvements in ADL scores were largely supported by the integration of exercise training and supervised nursing. Aerobic exercise, resistance training, and respiratory muscle training synergistically enhanced limb function and muscle strength, while supervised nursing ensured that exercise intensity remained safe and appropriate through dynamic follow-up. In addition, the family support component emphasized by IFSMT enabled family members to participate in supervision and assistance, further improving patients’ independence and safety in daily activities such as feeding and mobility (36). These functional improvements are closely associated with enhanced quality of life, which reflects the combined effects of physiological, psychological, and social interventions (22). The integrated intervention model—linking theory, nursing, and rehabilitation—not only highlights the guiding value of IFSMT in self-management but also underscores the regulatory role of supervised nursing and the physiological benefits of moderate-intensity exercise training, providing an effective pathway for improving quality of life in patients with stable COPD.

### Implications for practice

The comprehensive intervention integrating the Individual and Family Self-Management Theory (IFSMT), supervised nursing, and moderate-intensity exercise training provides an innovative and practical approach to pulmonary rehabilitation for patients with stable COPD. By implementing a context–process–outcome closed-loop framework, the intervention addresses key issues such as insufficient self-management, pronounced fatigue, and impaired pulmonary function. Contextual interventions shift patients and families from “passive acceptance” to “active engagement,” thereby enhancing adherence; process interventions, including dynamic supervision and individualized exercise plans, ensure the feasibility of implementation; while family support and emotional regulation complement traditional rehabilitation limitations, achieving multidimensional recovery. This approach not only improves pulmonary function and exercise tolerance but also facilitates the establishment of long-term health management habits and reduces the risk of acute exacerbations, providing a reference for the development of an integrated “hospital–community–home” management system. However, the implementation of this intervention may increase demands on hospital manpower, resources, and time, and should therefore be considered in light of the practical conditions of different institutions.

## Conclusions and recommendations

### Conclusion

The comprehensive intervention combining supervised nursing and moderate-intensity exercise training under the framework of the Individual and Family Self-Management Theory (IFSMT) represents an effective pulmonary rehabilitation strategy for patients with stable COPD. Through a randomized controlled trial, this study demonstrated that the intervention enhanced patients’ self-management ability, reduced fatigue, improved pulmonary function, exercise capacity and activities of daily living, and mitigated the overall impact of the disease on daily life, thereby comprehensively improving quality of life. These findings provide robust empirical evidence supporting the effectiveness of this integrated intervention and extend the application of IFSMT to the field of respiratory chronic disease rehabilitation, overcoming the limitations of traditional single-component interventions.

### Recommendations

It is recommended that this intervention be incorporated into routine clinical nursing practice in respiratory departments through the development of standardized operational protocols. Multidisciplinary IFSMT-based pulmonary rehabilitation teams—comprising respiratory physicians, rehabilitation therapists, and clinical nurses—should be established, with clearly defined roles and collaborative mechanisms. Core elements of IFSMT, supervised nursing skills, and individualized exercise training programs should be integrated into continuing professional education for healthcare providers, using diverse training modalities to enhance professional competence, particularly in exercise intensity assessment and adjustment. In addition, the intervention could be further optimized by integrating community and home-based care settings. Where feasible, community-based COPD pulmonary rehabilitation guidance centers could be established, and online rehabilitation management platforms could be developed to facilitate seamless coordination between hospital-based and home-based rehabilitation. In addition, strengthening training and guidance for family members through health education lectures and one-to-one demonstrations may enhance their ability to support disease management and supervision, enabling them to serve as an important source of long-term rehabilitation support and thereby consolidating the effects of the intervention.

### Limitations

This study has several limitations. First, it was a single-center trial conducted in a tertiary hospital, where medical resources (such as professional rehabilitation therapists and exercise equipment) are relatively abundant. This setting differs from that of primary care facilities or community health centers, which may limit the external validity of the results. However, most of the multidisciplinary team in our intervention was responsible for supervision and quality control, while the actual exercises were performed independently by patients. The intervention requires minimal hardware, and primary care institutions can adopt individual modules—such as follow-up, respiratory training, and family-assisted care—based on local resources. Second, the 3-month intervention period does not provide evidence for the long-term rehabilitation effects or sustainability of the program in COPD patients. Therefore, the durability of the observed benefits remains to be verified through longer-term follow-up studies. Third, due to objective constraints, no component-specific control groups were included, preventing the quantification of the independent effects of IFSMT-based guidance, supervised nursing, family support, and moderate-intensity exercise training, which may limit precise optimization of the intervention. Future studies may employ factorial designs or additional component-specific comparison groups to examine the relative contribution of each intervention component and further optimize the intervention model. Additionally, factors such as patients’ economic conditions, family support, and regional cultural differences were not considered, and the costs of implementing the intervention were not evaluated, which restricts the generalizability of recommendations. Future studies should explore multi-center designs, extended follow-up periods, additional control groups, and stratified analyses of confounding factors to enhance the value and applicability of the findings.

## Data Availability

The original contributions presented in the study are included in the article/supplementary material, further inquiries can be directed to the corresponding author.
